# Role of Nedd4L in Macrophage Pro-Inflammatory Polarization Induced by Influenza A Virus and Lipopolysaccharide Stimulation

**DOI:** 10.3390/microorganisms12071291

**Published:** 2024-06-25

**Authors:** Meihong Peng, Cheng Zhao, Fangguo Lu, Xianggang Zhang, Xiaoqi Wang, Li He, Bei Chen

**Affiliations:** 1 Medical School, Hunan University of Chinese Medicine, Changsha 410208, China; 20213696@stu.hnucm.edu.cn (M.P.); 20213695@stu.hnucm.edu.cn (L.H.); 20223769@stu.hnucm.edu.cn (B.C.); 2 School of Integrated Chinese and Western Medicine, Hunan University of Chinese Medicine, Changsha 410208, China; 20212095@stu.hnucm.edu.cn (C.Z.); 20222126@stu.hnucm.edu.cn (X.Z.); 20232123@stu.hnucm.edu.cn (X.W.)

**Keywords:** influenza A virus, bacterial co-infections, lipopolysaccharide, synergistic effects, Nedd4L expression, inflammatory response

## Abstract

Influenza A virus (IAV) infection often leads to influenza-associated fatalities, frequently compounded by subsequent bacterial infections, particularly Gram-negative bacterial co-infections. Lipopolysaccharide (LPS), a primary virulence factor in Gram-negative bacteria, plays a crucial role in influenza–bacterial co-infections. However, the precise pathogenic mechanisms underlying the synergistic effects of viral–bacterial co-infections remain elusive, posing significant challenges for disease management. In our study, we administered a combination of IAV and LPS to mice and examined associated parameters, including the lung function, lung index, wet/dry ratio, serum inflammatory cytokines, Nedd4L expression in lung tissue, and mRNA levels of inflammatory cytokines. Co-infection with IAV and LPS exacerbated lung tissue inflammation and amplified M1 macrophage expression in lung tissue. Additionally, we stimulated macrophages with IAV and LPS in vitro, assessing the inflammatory cytokine content in the cell supernatant and cytokine mRNA expression within the cells. This combined stimulation intensified the inflammatory response in macrophages and upregulated Nedd4L protein and mRNA expression. Subsequently, we used siRNA to knockdown Nedd4L in macrophages, revealing that suppression of Nedd4L expression alleviated the inflammatory response triggered by concurrent IAV and LPS stimulation. Collectively, these results highlight the pivotal role of Nedd4L in mediating the exacerbated inflammatory responses observed in IAV and LPS co-infections.

## 1. Introduction

Influenza, an acute respiratory disease, is caused by infection with the influenza virus, which is categorized into three types: A, B, and C, with type A infections being the most severe [1]. Influenza A virus (IAV), a member of the *Orthomyxoviridae* family, is an RNA virus that primarily causes acute upper respiratory tract infections and is rapidly transmitted through the air, potentially leading to global pandemics. Annually, there are approximately 1 billion cases of seasonal influenza, including 3–5 million severe cases, resulting in 290,000–650,000 respiratory-related deaths [2]. Generally, IAV infections trigger bronchitis and pneumonia with mild inflammation; however, co-infection with bacteria can lead to severe inflammatory responses in the lungs [3]. Studies suggest that many influenza-associated fatalities are due to mixed or secondary bacterial infections, with dysregulated host immune responses to these co-infections being a key factor in severe pneumonia outcomes [4].

In recent years, the increasing prevalence of Gram-negative bacteria in hospital-acquired infections (57.14%) and their growing antibiotic resistance have raised concerns over secondary Gram-negative bacterial infections following IAV infection. Lipopolysaccharide (LPS) is a major component of the outer membrane of Gram-negative bacteria and a key virulence factor that serves as a pathogen-associated molecular pattern for these bacteria [5,6]. In Gram-negative bacterial infections, bacteria and LPS stimulate macrophages in the host, altering their phenotype and promoting the expression of inflammatory cytokines, which, in turn, cause lung inflammation and injury.

During IAV infection, the virus first infects the mucosal epithelial cells, which then activates a macrophage-mediated immune response [7,8]. Macrophages are capable of internalizing and degrading pathogens via lysosomal pathways and present antigens to T cells that facilitate adaptive immune responses. They are likely to be among the first cells to respond to IAV infection, with their response being crucial for disease outcomes [9,10]. Macrophages display significant heterogeneity and rapidly alter their phenotype and function in response to host stimuli. Upon activation, resting macrophages transform into either the M1 or M2 phenotype. M1 macrophages possess phagocytic abilities against viruses and enhance the inflammatory response by presenting antigens to other cells, whereas M2 macrophages help to maintain homeostasis by secreting anti-inflammatory factors [11,12]. Excessive expression of inflammatory cytokines and chemokines by macrophages is one of the primary causes of the pathological damage caused by IAV infection. The severity of pathologic damage from viral–bacterial co-infections is closely linked to intense immune cell infiltration and the overexpression of pro-inflammatory factors [13]. The hyper-inflammatory response following secondary bacterial infections is a leading cause of high mortality in bacterial pneumonia following influenza infection. However, the pathogenic mechanisms underlying the synergistic effects of viral and bacterial co-infections remain unclear, presenting challenges for disease prevention and treatment.

Neural precursor cell-expressed, developmentally downregulated protein 4-like (Nedd4L) is highly expressed in macrophages. During IAV infection, Nedd4L regulates the expression of inflammatory cytokines to exert its antiviral effects. It also inhibits the transformation of M2 macrophages by downregulating TGF-β, thereby disturbing the balance of anti-inflammatory responses and impeding tissue repair functions [14,15]. However, the role of Nedd4L in co-infections with IAV and bacteria remains elusive. In this study, we hypothesized that under IAV + LPS stimulation, Nedd4L may exacerbate inflammatory responses by promoting the expression of pro-inflammatory cytokines or suppressing anti-inflammatory factors, leading to severe lung injury. We also explored the impact of Nedd4L in the context of lung injury under IAV + LPS stimulation and its mechanisms in macrophages.

## 2. Materials and Methods

### 2.1. IAV

The mouse-adapted IAV strain (A/PR/8/34) was kindly provided by the Virus Research Laboratory of Hunan Normal University (Changsha, China). This strain was cultured in the allantoic cavity of 10-day-old chicken embryos to achieve a hemagglutination titer of 1:640, which was suitable for use in the experiments.

### 2.2. Animal Source and Establishment of Model

Balb/c mice (6–8 weeks old, 20 ± 2 g) were procured from Hunan Slaccas Jinda Experimental Animal Co., Ltd. (Changsha, China), authorized under the license number SCXK(Xiang)2019-0004, with the experimental unit’s permit number SYXK(Xiang)2020-0010. Ethical handling of the mice was approved by the Animal Ethics Committee of the First Affiliated Hospital of Hunan University of Chinese Medicine (Changsha, China) (approval document number: ZYFY20230711-03). Sixty-four BALB/c mice, equally distributed between sexes, were acclimatized before being randomly divided into four groups: normal control (NC), IAV, LPS, and IAV + LPS, with eight mice per group. The IAV and IAV + LPS groups were inoculated intranasally with 50 µL of 50LD50 influenza virus to establish an infection model. Twenty-four hours post-infection, the LPS and IAV + LPS groups received an intranasal dose of LPS (1 mg/kg; L8880, Solarbio, Beijing, China). Pulmonary function tests and sample collections were conducted on four mice from the NC, IAV, LPS, and IAV + LPS groups at 6 h and 24 h post LPS intervention. The remaining four mice in each group were directly processed for sample collection.

### 2.3. Cell Culture, Transfection, and siRNA Silencing

RAW264.7 cells were obtained from the Cell Center of Xiangya Medical College (Changsha, China) and cultured in Dulbecco’s modified Eagle’s medium (DMEM; Gibco, Waltham, MA, USA) supplemented with 10% fetal bovine serum (FBS; Procell Life Science & Technology Co., Ltd., Wuhan, China). Nedd4L silencing was performed using small interfering RNAs (siRNAs; HANBio), which were transfected into cells using RNAfit (HANBio).

### 2.4. Establishment of Cell Model

Cells were resuspended in DMEM with 10% FBS and divided into the NC, IAV, LPS, and IAV + LPS groups. For IAV and IAV + LPS, a 100-fold dilution of the virus was introduced, followed by a 4 h incubation before switching to maintenance medium. After 24 h, LPS (1 μg/mL; Sigma L4391, Escherichia coli O111:B4, Sigma Chemical Co., St. Louis, MO, USA) was added to the LPS and IAV + LPS cells, followed by collection of supernatants and cells after 3 h/24 h.

### 2.5. Pulmonary Function

Mice were anesthetized with sodium pentobarbital (30 mg/kg) and intubated after tracheal exposure. The other end of the intubation tube was connected to a small animal pulmonary function tester (Harvard Bioscience Inc., DSI Buxco PFT, Holliston, MA, USA) to assess pulmonary function.

### 2.6. Lung Index and Wet-to-Dry Weight Ratio

The mouse body and lung masses were routinely measured, and the lung index and wet-to-dry weight ratio were calculated as follows:

Lung index(%) = Lung mass(g)/Body mass(g)*100%.Lung masses were recorded as wet weights, and lungs were then dried in a 65 °C oven for 24 h before measuring the dry weight. The wet-to-dry weight ratio was calculated as Wet-to-dry weight ratio = Wet lung weight(g)/Dry lung weight(g).

### 2.7. Hematoxylin and Eosin Staining of Mouse Lung Tissues

Mouse lung tissues were fixed in 4% paraformaldehyde (Biosharp, 22319084, Beijing, China) (for 1 week), followed by dehydration, clearing, and embedding in xylene. Tissues were sectioned into 5 μm thick slices, deparaffinized, stained, and observed under a light microscope after rehydration and sealing.

### 2.8. Enzyme-Linked Immunosorbent for Mouse Serum and Cell Culture Supernatants

The levels of inflammatory cytokines in the serum and cell culture supernatants were measured using IL-6/TNF-α/TGF-β/IL-10 ELISA kits (Shanghai Enzyme-linked Biotechnology Co., Ltd., Shanghai, China). After incubation and washing, a substrate solution was added and the reaction was stopped for optical density measurement at 450 nm using a spectrophotometer.

### 2.9. Immunohistochemistry for Nedd4L Protein Expression in Mouse Lung Tissues

Mouse lung tissues fixed in 4% paraformaldehyde were processed as described above and stained with Nedd4L antibodies and secondary horseradish peroxidase (HRP)-conjugated antibodies. After staining and sealing, lung tissues were examined under a light microscope to assess Nedd4L protein expression.

### 2.10. Immunofluorescence for CD86 and CD206 Protein Expression in Mouse Lung Tissues

Lung tissues prepared as described above were subjected to antigen retrieval, serum blocking, and overnight incubation with primary antibodies against CD86 (abcam, ab119857, Cambridge, UK) and CD206 (abcam, ab64693) at 4 °C. After washing, the tissues were incubated with goat anti-rabbit IgG (Adobe 1:40,000) for 2 h, re-washed, and examined under a fluorescence microscope in the dark.

### 2.11. Real-Time Quantitative PCR for Nedd4L and Inflammatory Cytokine MRNA Expression in Mouse Lung Tissues and Macrophages

RNA from lung tissues and cells was extracted using a reagent kit (Tiangen Total RNA, DP149, Beijing, China), converted to cDNA using a reverse transcription kit (NovoStart^®^ cDNA SuperMix Plus, E047-01B, Suzhou, China), and amplified using a qPCR kit (NovoStart^®^ SYBR qPCR SuperMix Plus, E099-01A, Suzhou, China).

### 2.12. Western Blotting for Nedd4L Protein Expression in Cells

Cellular proteins were prepared with RIPA and 5× loading buffer, subjected to electrophoresis, transferred to membranes, blocked, and incubated with primary antibody (1:1000; Nedd4L, Protechtein, 13690-1-AP, Wuhan, China) overnight at 4 °C. The bands were visualized by Bio-Rad Mini-Protean PowerPac after washing and incubation with secondary antibodies.

### 2.13. Statistical Analysis

The experimental data were statistically analyzed using SPSS 25.0 software, and graphically analyzed using GraphPad Prism 8.0. Results were presented as mean ± standard deviation (x ± s). Normally distributed data were analyzed using ANOVA, whereas non-parametric tests were used for non-normally distributed data. Statistical significance was set at *p* < 0.05.

## 3. Results

### 3.1. Impact of Combined IAV and LPS Stimulation on Mouse Pulmonary Function

**Figure 1:**
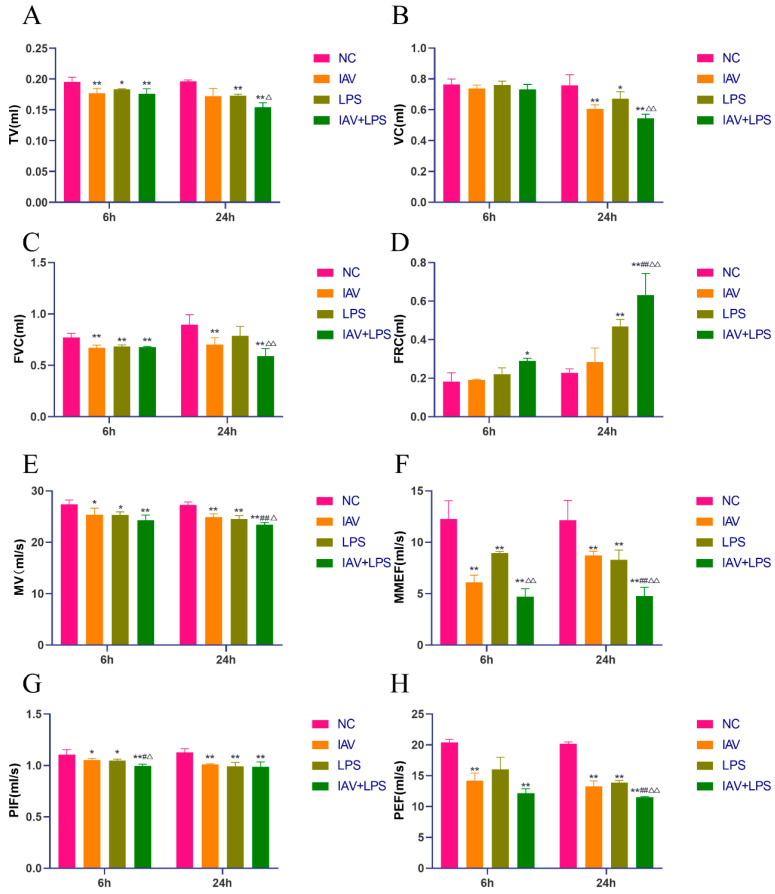
Impact of IAV + LPS on mouse pulmonary function. (**A**–**H**) BALB/c mice were treated with saline, IAV, LPS, and IAV + LPS. Mice were anesthetized at 6 h/24 h post-LPS intervention, and pulmonary function was assessed using a small animal lung function tester. (**A**) Tidal volume (TV) (*n* = 4). (**B**) Functional residual capacity (FRC) (*n* = 4). (**C**) Vital capacity (VC) (*n* = 4). (**D**) Forced vital capacity (FVC) (*n* = 4). (**E**) Minute volume (MV) (*n* = 4). (**F**) Maximal midexpiratory flow (MMEF) (*n* = 4). (**G**) Peak inspiratory flow (PIF) (*n* = 4). (**H**) Peak expiratory flow (PEF) (*n* = 4). Compared with the NC group: * *p* < 0.05, ** *p* < 0.01; compared with the IAV group: ^#^ *p* < 0.05, ^##^ *p* < 0.01; compared with the LPS group: ^△^ *p* < 0.05, ^△△^ *p* < 0.01.

Our analysis of pulmonary function parameters across various groups revealed that the IAV/LPS/IAV + LPS groups exhibited a reduced tidal volume (TV), vital capacity (VC), and forced vital capacity (FVC) relative to the NC group. The reductions in TV, VC, and FVC were more pronounced in the IAV + LPS group than in the IAV and LPS groups (Figure 1A–C). Additionally, the functional residual capacity (FRC) increased to varying degrees across all interventions, with the most significant increase observed in the IAV + LPS group (Figure 1D). Both IAV and LPS interventions independently caused a decline in pulmonary ventilation function, which was further exacerbated by combined IAV + LPS stimulation. Furthermore, we assessed lung-volume-related parameters such as minute ventilation volume (MV), maximal midexpiratory flow (MMEF), peak inspiratory flow (PIF), and peak expiratory flow (PEF). All samples showed reductions in these parameters, with the most substantial decline observed in the IAV + LPS group (Figure 1E–H). These findings indicate that both IAV and LPS interventions impair lung volume functions, leading to reduced lung volumes, which, in turn, decreases alveolar ventilation and results in lower venous blood oxygen levels, ultimately resulting in damage to the lungs and potentially to the whole organism. In conclusion, the combined IAV + LPS intervention aggravated pulmonary ventilation and lung volume impairment, thereby exacerbating lung function injury.

### 3.2. Damage and Edema in Mouse Lung Tissue Due to Combined IAV and LPS Stimulation

**Figure 2:**
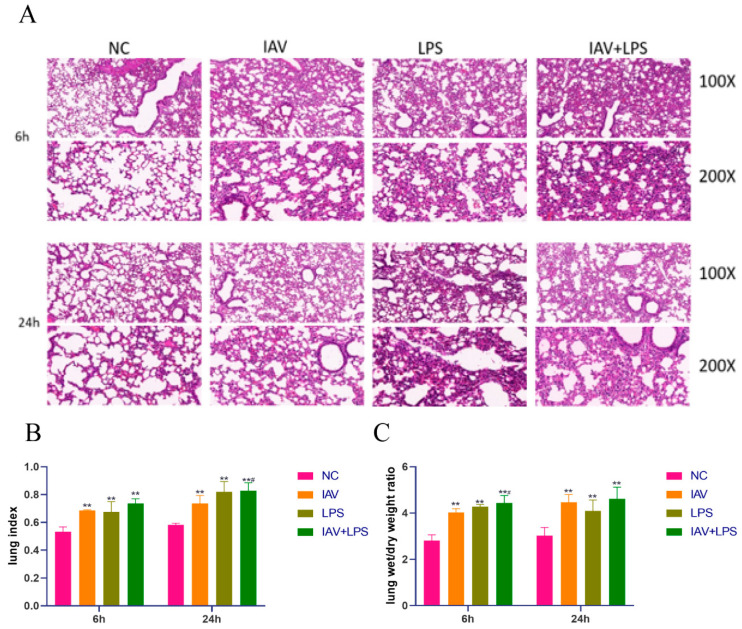
Lung tissue damage, increased lung index, and wet-to-dry weight ratio due to IAV + LPS. (**A**–**C**) BALB/c mice were treated with saline, IAV, LPS, and IAV + LPS. Mice were anesthetized 6 h/24 h post-LPS intervention. Lung tissues were fixed, stained with hematoxylin and eosin (H&E), and examined under a light microscope at 100× and 200× magnifications. Body mass, wet lung weight, and dry lung weight were routinely measured, and both the lung index and wet-to-dry weight ratio were calculated. (**A**) H&E staining of lung tissue. (**B**) Lung index calculated as lung wet weight (g)/body mass (g) (*n* = 4). (**C**) Wet-to-dry weight ratio calculated as lung wet weight (g)/lung dry weight (g) (*n* = 4). Compared with the NC group: ** *p* < 0.01; compared with the IAV group: ^#^ *p* < 0.05.

Examination of mouse lung tissue sections under a light microscope revealed pathological changes in all intervention groups, including alveolar collapse, thickening of interstitial lung tissue, increased infiltration of inflammatory cells, pulmonary edema, and congestion. These effects were more pronounced in the IAV + LPS group, which exhibited increased inflammatory cell infiltration, thickening of alveolar walls, alveolar edema, necrosis, and hemorrhage compared to the solely IAV and LPS groups (Figure 2A). Additionally, when analyzing the lung index, a measure of pulmonary injury, we found an increase across all interventions compared to the normal group. Notably, the increase in the lung index was more significant in the IAV + LPS group at 24 h post-intervention (Figure 2B). A similar trend was observed in the wet-to-dry weight ratio of the lungs, with the IAV + LPS group showing a more marked increase at 6 h post-intervention than that of the IAV group (Figure 2C). These findings suggested that IAV + LPS stimulation exacerbated the lung tissue pathology, thereby intensifying lung damage. The increase in both the lung index and wet-to-dry weight ratio also indicates that both IAV and LPS can cause pulmonary edema, with the combined IAV + LPS intervention further aggravating this condition.

### 3.3. Effects of Combined IAV and LPS Stimulation on Serum Inflammatory Cytokines and Lung Tissue Inflammatory Cytokine mRNA Expression in Mice

**Figure 3:**
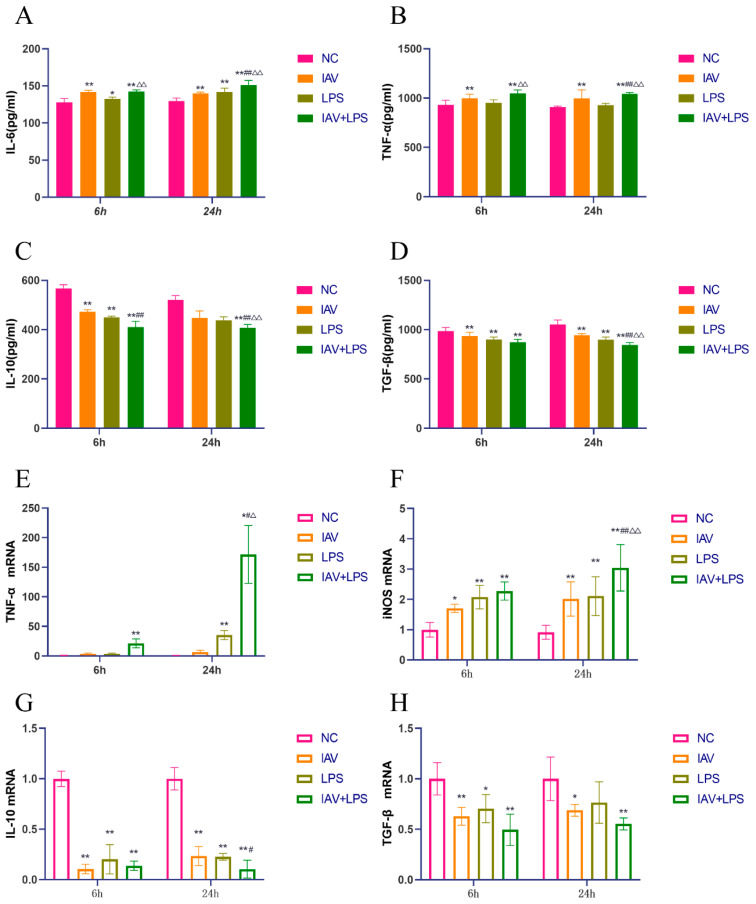
Effects of IAV + LPS on the secretion of inflammatory and anti-inflammatory cytokines in mouse serum and on the mRNA expression of inflammatory cytokines in lung tissue. BALB/c mice were treated with saline, IAV, LPS, and IAV + LPS. Mice were anesthetized 6 h/24 h post-LPS intervention, and samples of mouse serum and lung tissue were collected. We observed: (**A**–**D**) serum levels of the inflammatory cytokines IL-6, TNF-α, IL-10, and TGF-β (pg/mL) (*n* = 6). (**E**–**H**) mRNA expression of the inflammatory cytokines TNF-α, iNOS, IL-10, and TGF-β in lung tissue (*n* = 4). Compared with the NC group: * *p* < 0.05, ** *p* < 0.01; compared with the IAV group: ^#^ *p* < 0.05, ^##^ *p* < 0.01; compared with the LPS group: ^△^ *p* < 0.05, ^△△^ *p* < 0.01.

From the lung index and wet-to-dry weight ratio results, it was evident that IAV + LPS stimulation led to significant pulmonary edema, with pathological sections revealing the infiltration of inflammatory cells in the lungs. Subsequently, we analyzed inflammatory cytokines in mouse serum and observed a marked increase in the levels of pro-inflammatory cytokines IL-6 and TNF-α across all interventions, with the most significant upregulation in the IAV + LPS group (Figure 3A,B). Meanwhile, anti-inflammatory cytokines IL-10 and TGF-β showed downregulation, particularly in the IAV + LPS group (Figure 3C,D). Additionally, we assessed the mRNA expression of inflammatory cytokines in the lung tissue and found trends consistent with serum cytokine levels. The expression of pro-inflammatory cytokine mRNAs such as TNF-α and iNOS was elevated across all interventions, with the most substantial increases observed in the IAV + LPS group (Figure 3E,F). The mRNA expression of anti-inflammatory cytokines IL-10 and TGF-β decreased, with the most significant reductions also associated with the IAV + LPS group (Figure 3G,H). These findings indicate that IAV or LPS can induce increases in serum pro-inflammatory cytokines and decreases in anti-inflammatory cytokines, as well as the upregulation of pro-inflammatory cytokines and downregulation of anti-inflammatory cytokine expression in lung tissue. Combined IAV + LPS stimulation exacerbated these effects, intensifying the pulmonary inflammatory response and leading to more severe inflammatory lung damage.

### 3.4. Impact of Combined IAV and LPS Stimulation on Macrophage Phenotypes in Lung Tissue

**Figure 4:**
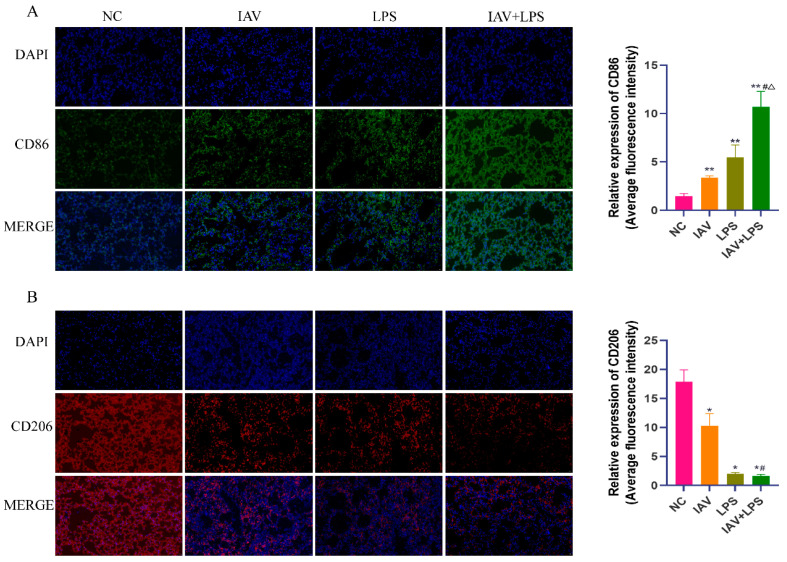
IAV + LPS induces increased expression of CD86 and decreased expression of CD206 in mouse lung tissue. (**A**,**B**) BALB/c mice were treated with saline, IAV, LPS, and IAV + LPS. Mice were anesthetized 24 h after LPS intervention, and lung tissues were subjected to immunofluorescence staining for CD86 and CD206. (**A**) Immunofluorescence staining for CD86 in lung tissue (*n* = 3). (**B**) Immunofluorescence staining for CD206 in lung tissue (*n* = 3). Compared with the NC group: * *p* < 0.05, ** *p* < 0.01; compared with the IAV group: ^#^ *p* < 0.05; compared with the LPS group: ^△^ *p* < 0.05.

Macrophages are primary immune cells in the lungs and play a crucial role in the response to viral and bacterial infections. Owing to their heterogeneity, macrophages can transform into different phenotypes under various stimuli to fulfill specific functions. Our earlier experimental results indicated that injury from the combined effect of IAV and LPS at 24 h was more severe than that at 6 h. Consequently, we examined the lung tissues of mice 24 h after IAV + LPS stimulation via immunofluorescence staining for CD86 and CD206. We found that the combined IAV + LPS stimulation led to an increase in the expression of the M1 macrophage surface molecule CD86 and a decrease in the expression of the M2 macrophage surface molecule CD206 (Figure 4A,B). This suggests that IAV + LPS stimulation promotes an increase in lung M1 macrophages, which are primarily pro-inflammatory cells, consistent with our previous findings. Based on these observations, we hypothesized that IAV + LPS exacerbated lung tissue inflammation and subsequent damage by promoting the conversion of lung macrophages to the M1 phenotype.

### 3.5. Impact of Combined IAV and LPS Stimulation on Macrophage Inflammatory Cytokine Expression

**Figure 5:**
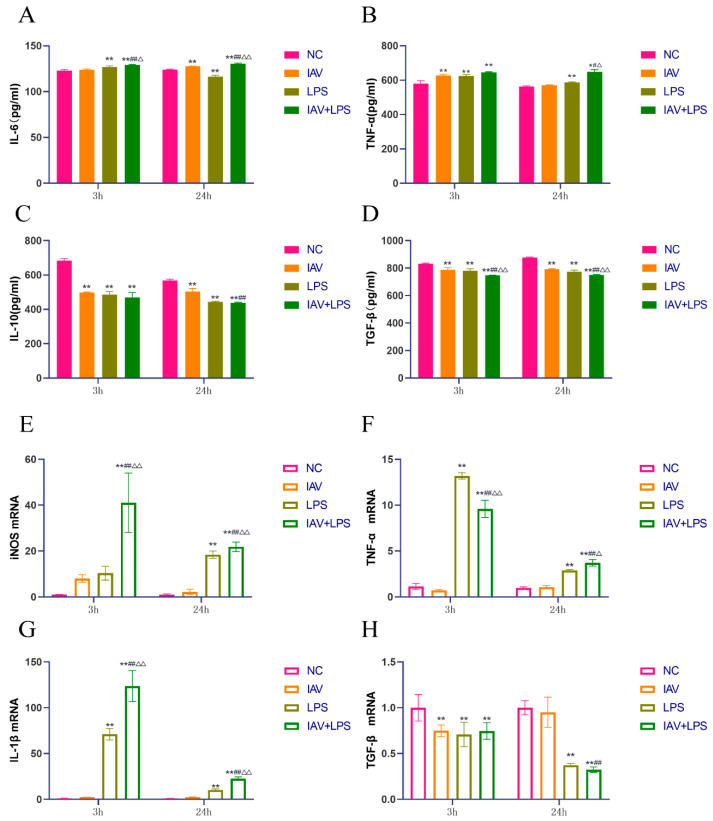
Impact of IAV + LPS stimulation on macrophage pro-inflammatory and anti-inflammatory cytokine secretion and expression. RAW264.7 cells were treated with 10% fetal bovine serum (FBS) in Dulbecco’s modified Eagle’s medium (DMEM), IAV, LPS, and IAV + LPS. Cells and supernatants were collected 3 h/24 h after LPS intervention for analysis. Cytokine levels in the cell culture supernatants were quantified via ELISA, and cellular mRNA expression levels were assessed via RT-PCR. (**A**–**D**) IL-6, TNF-α, IL-10, and TGF-β levels in cell supernatant (pg/mL) (*n* = 3). (**E**–**H**) iNOS, TNF-α, IL-1β, and TGF-β mRNA expression in cells (*n* = 3). Compared with the NC group: * *p* < 0.05, ** *p* < 0.01; compared with the IAV group: ^#^ *p* < 0.05, ^##^ *p* < 0.01; compared with the LPS group: ^△^ *p* < 0.05, ^△△^ *p* < 0.01.

In vivo experiments in mice demonstrated that IAV + LPS stimulation promoted the transformation of lung tissue macrophages toward the M1 phenotype and reduced their transformation to the M2 phenotype. To investigate the primary mechanisms by which IAV + LPS affects pulmonary inflammation and the role of macrophages, we utilized RAW264.7 cells to further study the impact of IAV + LPS on the secretion and expression of macrophage cytokines. The results indicated that the levels of IL-6 and TNF-α in the cell culture supernatants increased across all interventions, with the increase being more pronounced in the IAV + LPS group compared to the solely IAV or LPS group (Figure 5A,B). Similarly, the levels of IL-10 and TGF-β in the supernatants decreased, with the most significant reductions observed in the IAV + LPS group (Figure 5C,D). The mRNA expression levels of inflammatory cytokines in the cells mirrored the trends observed in the culture supernatants, with increased expression of pro-inflammatory cytokines iNOS, TNF-α, and IL-1β mRNA (Figure 5E–G) and decreased expression of the anti-inflammatory cytokine TGF-β mRNA (Figure 5H). These findings suggest that both IAV and LPS interventions independently affect macrophage cytokine secretion and mRNA expression, with the combined IAV + LPS intervention further enhancing these effects.

### 3.6. Impact of Combined IAV and LPS Stimulation on Nedd4L

**Figure 6:**
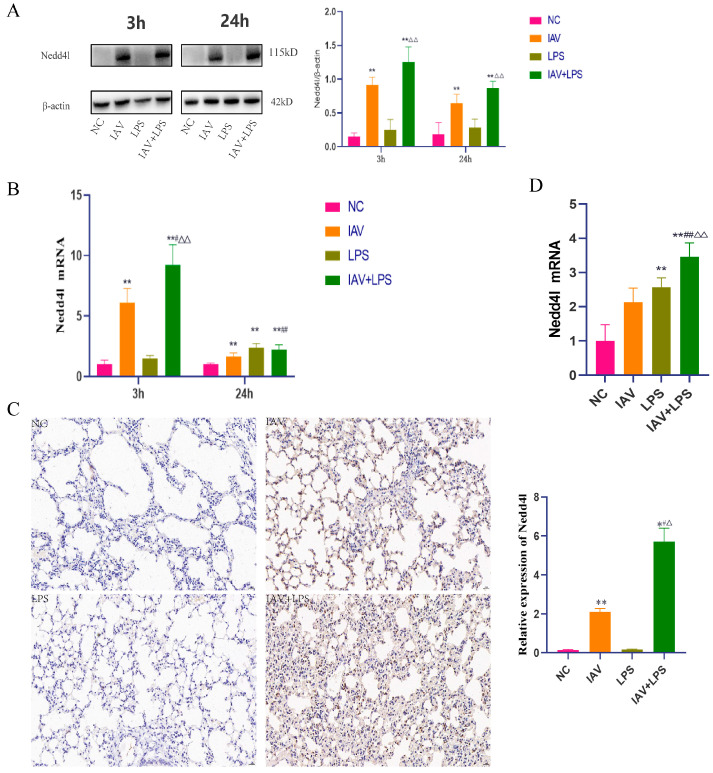
Increased Nedd4L protein and mRNA expression in macrophages and mouse lung tissue following IAV + LPS stimulation. (**A**,**B**) RAW264.7 cells were treated with 10% FBS in DMEM, IAV, LPS, and IAV + LPS. Cells were collected 3 h/24 h after LPS intervention, and Nedd4L protein expression was analyzed via Western blotting. (**A**) Nedd4L protein expression in cells (*n* = 3). (**B**) Nedd4L mRNA expression in cells (*n* = 3). (**C**,**D**) BALB/c mice were treated with saline, IAV, LPS, and IAV + LPS. Mice were euthanized 24 h post-intervention, lung tissues were collected, and Nedd4L protein expression was assessed via immunohistochemistry, while Nedd4L mRNA expression was evaluated via RT-PCR. (**C**) Nedd4L protein expression in lung tissue (*n* = 3). (**D**) Nedd4L mRNA expression in lung tissue (*n* = 3). Compared with the NC group: * *p* < 0.05, ** *p* < 0.01; compared with the IAV group: ^#^ *p* < 0.05, ^##^ *p* < 0.01; compared with the LPS group: ^△^ *p* < 0.05, ^△△^ *p* < 0.01.

Previous studies have established that both IAV and LPS influence the expression of inflammatory cytokines in macrophages, with LPS exacerbating the IAV-induced inflammatory response. Nedd4L is a naturally occurring antiviral immune factor highly expressed in macrophages. Analysis of Nedd4L mRNA expression in macrophages revealed that IAV infection promoted Nedd4L mRNA expression, and LPS stimulation further increased its expression (Figure 6A). Additionally, the protein expression analysis of Nedd4L in these cells showed an increase following IAV infection, which was further enhanced by LPS stimulation (Figure 6B). Given the earlier in vivo findings that combined stimulation for 24 h resulted in more significant lung tissue damage and inflammatory response than stimulation for 6 h, we also examined the protein expression and localization of Nedd4L in the lung tissue after 24 h of combined stimulation. This investigation showed an increase in Nedd4L protein expression in lung tissues (Figure 6C). Further examination of Nedd4L mRNA expression in the lung tissues after 24 h of combined stimulation revealed that its expression in the IAV + LPS group was significantly higher than that in either the solely IAV or LPS group (Figure 6D). These findings indicate that combined IAV and LPS stimulation enhances Nedd4L expression in both lung tissue and macrophages. Given Nedd4L’s role in regulating macrophage phenotypes and the secretion of inflammatory cytokines, it is plausible to hypothesize that the exacerbation of the inflammatory response in the IAV + LPS group might be due to the increased expression of Nedd4L.

### 3.7. Impact of Nedd4L Silencing on Cytokine Expression in Macrophages Stimulated with Combined IAV and LPS

**Figure 7:**
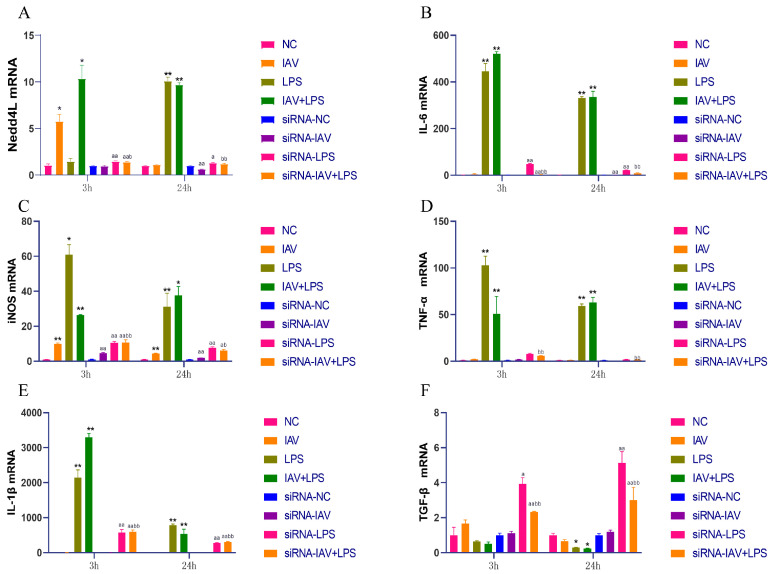
Effects of Nedd4L siRNA-mediated silencing on cytokine expression in RAW264.7 macrophages stimulated with IAV + LPS. RAW264.7 cells were treated with 10% FBS in DMEM, IAV, LPS, and IAV + LPS following Nedd4L siRNA treatment for 12 h. Cells were collected 3 h/24 h after LPS intervention, and RT-PCR was used to assess the expression of Nedd4L, IL-6, iNOS, TNF-α, IL-1β, and TGF-β mRNA. (**A**–**F**) Nedd4L, IL-6, iNOS, TNF-α,IL-1β, and TGF-β mRNA expression in cells (*n* = 3). Compared with the NC group: * *p* < 0.05, ** *p* < 0.01; compared with the siRNA-NC group: ^a^ *p* < 0.05, ^aa^ *p* < 0.01; compared with the IAV + LPS group: ^b^ *p* < 0.05, ^bb^ *p* < 0.01.

Our results showed that Nedd4L expression was enhanced in macrophages and upregulated in lung tissues. Therefore, we used siRNA technology to silence Nedd4L in macrophages and examined the expression of Nedd4L mRNA (Figure 7A). The silencing of Nedd4L using siRNA suppressed Nedd4L mRNA expression to varying degrees. We also analyzed the mRNA expression of IL-6, iNOS, TNF-α, IL-1β, and TGF-β across the different treatment groups. Our results indicated that macrophages treated with IAV + LPS, followed by Nedd4L siRNA treatment, exhibited decreased expression of IL-6, iNOS, TNF-α, and IL-1β mRNA (Figure 7B–E), alongside an upregulation of TGF-β mRNA expression (Figure 7F). These findings suggest that Nedd4L expression’s regulation may mediate the inflammatory response induced by IAV + LPS stimulation in macrophages. LPS can promote the expression of inflammatory cytokines by mediating Nedd4L expression, leading to severe inflammatory responses. This suggests that the inflammatory response induced in macrophages by IAV + LPS stimulation may be mediated through the regulation of Nedd4L expression. LPS promotes the expression of inflammatory cytokines by mediating Nedd4L expression, leading to a severe inflammatory response.

## 4. Discussion

IAV infection affects the host antibacterial immune response, rendering the upper respiratory tract more susceptible to bacterial invasion, enhancing the colonization ability of bacteria in the lower respiratory tract, and leading to a decrease in host immunity. Research indicates that the sequence of viral and bacterial infections is crucial for disease progression, with the most severe damage and highest mortality occurring when bacterial infection follows viral infection. Post-influenza viral infection, due to the disruption of the pulmonary epithelial barrier, reduction in active substances, and increased expression of glycoprotein 96 (GP96) in lung epithelial cells, leads to heightened bacterial susceptibility. Concurrently, decreased G-CSF levels following influenza viral infection also facilitate the onset of secondary pneumonia [16,17]. Consequently, the pathogenicity of opportunistic pathogens in the host increases, thereby increasing the rate of bacterial infections following influenza viral infection [7,18]. During co-infection, the neuraminidase of IAV cleaves sialic acid residues, exposing receptors on the surface of upper respiratory tract cells, which facilitates bacterial invasion and increases the likelihood of secondary bacterial pneumonia [19,20,21]. Recruitment of immune cells to the lungs during secondary bacterial pneumonia, along with an increase in bacterial density, exacerbates pulmonary inflammation. Moreover, co-infection with influenza virus and bacteria is a major contributor to the acute exacerbation and increased mortality of chronic obstructive pulmonary disease [22,23]. Recent studies have shown an increase in hospital-acquired infections caused by Gram-negative bacteria, leading to more common cases of secondary Gram-negative bacterial pneumonia. Pathogens such as *Haemophilus influenzae*, *Pseudomonas aeruginosa*, and *Streptococcus pneumoniae* are the most common causative agents of influenza-associated bacterial pneumonia [24]. Following influenza viral infection, elevated levels of IFN, a crucial antibacterial immune factor, can increase susceptibility to Gram-negative bacteria by modulating IL-17 [25,26]. Additionally, the influenza virus can affect Toll-like receptor pathways, influencing the immune response. The secretion of inflammatory cytokines such as TNF-α, IL-1β, and IL-6 post influenza infection also promotes bacterial adhesion and invasion [27]. Our findings indicate that combined stimulation with IAV and LPS impairs lung function and increases the secretion of IL-6, TNF-α, iNOS, and IL-1β, major regulators of inflammatory responses, while simultaneously decreasing the secretion of IL-10 and TGF-β. This suggests that combined IAV and LPS stimulation not only promotes the inflammatory response but also inhibits anti-inflammatory responses and tissue repair, further exacerbating lung tissue injury.

Macrophages are key immune cells that play crucial roles in the response to combined IAV and LPS stimulation. Macrophages can transform into different phenotypes under various stimuli, and the balance between M1/M2 macrophage phenotypes is crucial for maintaining stability between tissue damage and repair [28,29]. However, in this study, the combination of IAV infection and LPS stimulation disrupted this balance. Post-stimulation, there was an increase in CD86^+^ macrophages and a decrease in CD206^+^ macrophages in the lung tissues. CD86^+^ is a major surface marker of M1 macrophages, which primarily promotes inflammatory responses, consistent with our analysis of inflammatory cytokines. M1 macrophages promote inflammation through the secretion of iIL-6, TNF-α, IL-1β, and iNOS, whereas M2 macrophages mainly mitigate inflammation and aid in tissue repair through the secretion of IL-10, TGF-β, and other anti-inflammatory factors.

Nedd4L is an important regulator of inflammatory responses, playing significant roles in both influenza virus and bacterial infections [30,31]. It can affect macrophage phenotype transformation by regulating cellular Na^+^ channels, which modulates cytokine secretion, and also by affecting the secretion of Smad factors, thus influencing TGF-β [32].

Secondary infection with *Pseudomonas aeruginosa* following the influenza virus can lead to exacerbated damage through downregulation of pulmonary inflammatory restoration [33], which is consistent with our research findings. Our results indicate that IAV + LPS stimulation promotes Nedd4L expression, leading to overexpression of IL-6, TNF-α, iNOS, and IL-1β in macrophages and suppression of TGF-β expression, resulting in a dysregulation of the inflammatory response and impacting tissue inflammation and repair. Silencing of Nedd4L in RAW264.7 cells, after IAV + LPS stimulation, confirmed the regulatory role of Nedd4L on inflammatory cytokines. With Nedd4L gene silencing, the expression of inflammatory cytokines IL-6, TNF-α, iNOS, and IL-1β was suppressed, while the expression of the anti-inflammatory cytokine TGF-β was upregulated. This indicates that Nedd4L not only exacerbates inflammatory responses by regulating pro-inflammatory cytokine secretion but also by suppressing anti-inflammatory cytokine secretion, further aggravating tissue damage. When Nedd4L was silenced in macrophages, the severity of the inflammatory response induced by IAV + LPS was mitigated.

In summary, this study proposes that stimulation with IAV + LPS induces severe inflammatory responses more significantly than stimulation with IAV or LPS alone by promoting Nedd4L expression, which disrupts the balance of M1/M2 macrophages. It does so by upregulating the secretion of pro-inflammatory factors and downregulating the secretion of anti-inflammatory factors, thereby leading to the aggravation of lung tissue damage.

## 5. Conclusions

This study demonstrates that Nedd4L exacerbates lung inflammation injury in mice subjected to combined exposure to influenza virus and LPS, accompanied by upregulated expression of Nedd4L in lung tissues. It also confirms that stimulation with both influenza virus and LPS promotes Nedd4L expression, subsequently upregulating the expression of macrophage inflammatory factors. However, this study does not delve into the specific mechanisms by which Nedd4L regulates inflammatory factors, and nor does it clarify the mechanism of action of Nedd4L in vivo in mice. In future studies, we will further explore the specific mechanisms through which Nedd4L regulates inflammatory factors under the dual stimulation of influenza virus and LPS. Understanding the mechanisms of damage caused by the combined stimulation of influenza virus and LPS can provide a preliminary research foundation for studies on secondary bacterial pneumonia following the influenza virus. This, in turn, can offer a certain theoretical basis for the prevention and treatment of secondary bacterial pneumonia post viral infection.

In this field of study, most current research on secondary bacterial pneumonia following the influenza virus focuses on Gram-positive bacteria, with studies on Gram-negative bacteria needing further supplementation. There is also a lack of well-established model references for secondary bacterial pneumonia following the influenza virus in in vivo experiments. Meanwhile, in in vitro experiments, when cell cultures are contaminated, the majority of contamination originates from bacteria. Therefore, the ways in which to effectively manage the use of bacteria in cell models require further exploration.

## Data Availability

Data are contained within the article.
